# Improvement of personality functioning among people treated within personality disorder mental health services. A longitudinal, observational study

**DOI:** 10.3389/fpsyt.2023.1163347

**Published:** 2023-05-09

**Authors:** Elfrida H. Kvarstein, Mathias Frøyhaug, Mona S. Pettersen, Sara Carlsen, Andreas Ekberg, Jane Fjermestad-Noll, Dag A. Ulvestad, Elisabeth L. Gikling, Eirik Hjermann, Kenneth Lindberget, Siri Omvik, Ingeborg U-M. Eikenæs, Benjamin Hummelen, Katharina T. E. Morken, Theresa Wilberg, Geir A. F. Pedersen

**Affiliations:** ^1^Section for Personality Psychiatry and Specialized Treatment, Oslo University Hospital, Oslo, Norway; ^2^Institute of Clinical Medicine, University of Oslo, Oslo, Norway; ^3^Groruddalen District Psychiatric Center, Akershus University Hospital, Akershus, Norway; ^4^Tromsø University Hospital, Tromsø, Norway; ^5^Department of Addiction Medicine, Haukeland University Hospital, Bergen, Norway; ^6^Department for Adult Psychiatry, Diakonhjemmet Hospital, Oslo, Norway; ^7^Kronstad District Psychiatric Center, Haukeland University Hospital, Bergen, Norway; ^8^Strømme District Psychiatric Center, Sørlandet Hospital, Kristiansand, Norway; ^9^Department of Research and Innovation, Oslo University Hospital, Oslo, Norway; ^10^Department of Clinical Psychology, University of Bergen, Bergen, Norway; ^11^Institute of Basic Medical Sciences, University of Oslo, Oslo, Norway

**Keywords:** personality disorder (MeSH), personality functioning, treatment, longitudinal, improvement

## Abstract

**Objective:**

Evidence-based personality disorder (PD) treatments are dominated by interventions targeting Borderline PD, although clinical populations characteristically include different PD features and severity. Personality functioning is a new concept intended to capture common features across PDs. This study aimed to investigate longitudinal improvement of personality functioning in a clinical sample assigned to PD treatment.

**Method:**

An observational, large, longitudinal study of patients in PD treatments on specialist mental health service levels (*N* = 1,051). DSM-5 PDs were systematically assessed on referral. Personality functioning was repeatedly assessed (LPFS-BF-2.0), supplemented by symptom distress (anxiety: PHQ-GAD-7, depression: PHQ-9), and social/occupational activity (WSAS, work/study activity). Statistics were linear mixed models.

**Results:**

Thirty per cent had personality difficulties below PD threshold. Among PDs, 31% had Borderline (BPD), 39% Avoidant (AvPD), 15% not otherwise specified, 15% other PDs, and 24% > one PD. More severe initial LPFS-BF was associated with younger age, presence of PD and increasing number of total PD criteria. Across PD conditions, LPFS-BF, PHQ-9 and GAD-7 improved significantly (overall effect size 0.9). Mean duration of PD treatment was 15 (SD 9) months. Drop-out rates were low (12%). LPFS-BF improvement-rates were higher for BPD. Younger age was moderately associated with slower PHQ-9 improvement. Work/study activity was initially poor, poorer levels associated with AvPD and younger age, and improvement was non-significant across PD conditions. AvPD was associated with slower WSAS improvement-rates.

**Conclusion:**

Personality functioning improved across PD conditions. The results highlight BPD improvements. The study points to challenges concerning AvPD treatment, poor occupational activity and age-related differences.

## Introduction

Personality disorders (PDs) are common and severe mental disorders associated with impaired quality of life (1). The adult population prevalence approximates 10% in high income countries (2). Within mental health services a prevalence of 40% is reported (3). Studies also document potentials for improvement over time - ranging from full PD recovery, PD trait remission, and more fluctuating or persistent vulnerability (4, 5). Borderline PD (BPD) is the most studied disorder and several treatment interventions are recommended (6–8). Nonetheless, in clinical practice, patients referred to treatment present a broader range of personality difficulties, disorders, and comorbidities (3, 9). Reports indicate that Avoidant (AvPD), PD not otherwise specified (PD-NOS), and comorbid PD conditions also represent frequent, clinically relevant conditions (10–14). Despite high clinical relevance, PD heterogeneity is, unfortunately, not well reflected in current evidence-base on treatment interventions. There is however, an increasing focus on PD severity across specific diagnostic categories (15, 16).

The quite recently established “Level of Personality Functioning Scale”(LPFS) in DSM-5 provides a means for grading personality problems (17). The concept of personality functioning is intended to represent the severity of core aspects common to different PD types or traits. As presented in the Alternative Model for Personality Disorders, the LPFS includes personality domains of identity, self-direction, empathy, and intimacy (18). Similarly, the ICD-11 classifies PD severity by a global assessment of self and interpersonal functioning (19). In both assessment systems, the overall dimensional evaluation of severity addresses issues of overlapping PD criteria, PD comorbidities, and clinically relevant subthreshold difficulties – considerations that are highly relevant in the typically heterogenic samples encountered in clinical practice.

Different methods for assessing personality functioning have been developed (20–26), and a brief LPFS self-report [LPFS-BF 2.0 (25)] has recently been included in a recommended, standard set of outcome measures for people with PD (multidisciplinary expert working group, International Consortium for Health Outcomes Measurement) (27). A current review emphasized associations between more impaired personality functioning, greater drop-out, and poorer treatment alliance and outcomes (28). The authors concluded that more research focusing on clinical implications of PD severity is needed (28). Assessment of personality functioning is a new development. Although the above-mentioned study of Weekers et al. (25) included investigation of LPFS-BF change over the course of a treatment program, there is still a lack of studies investigating how overall personality functioning develops over time in large, heterogenous clinical populations.

Treatment seeking patients with PDs often have high levels of symptom distress (10). Comorbid symptom disorders, such as anxiety and mood disorders, render a high subjective burden, and are frequently diagnosed. Their course is known to be complicated by persisting PD, but with PD remission, concomitant improvement of mental distress and symptom disorders is also described (29–32). Such parallel developments suggest an important interplay between mental distress and personality dysfunction (33–37). In studies of PD treatments, measures of symptom distress are usually included among outcomes (38–40). A considerable decline of mental distress is reported in BPD treatment trials (41–43). It is likely that improvement of personality functioning will be accompanied by improvement of comorbid symptom distress.

Social and occupational engagement is an important predictor of good health (44, 45) and should therefore also be a relevant outcome measure in clinical studies. Negative health effects of unemployment, such as longstanding depressive symptoms are emphasized in studies of older adults with BPD (46). Occupational functioning may vary among people with PDs. However, studies emphasize a relationship between more severe personality pathology and low educational level, unstable work attendance, conflicts in the workplace, increased chance of unemployment, and high rates of disability – the latter representing high societal costs (47–50). Severe occupational impairment is described in clinical BPD samples (51). Nonetheless, few treatment trials report such activity as an outcome. Some treatment studies suggest little effect on work functioning for patients with BPD (52–54), though better in a long-term perspective (55–57). In general, few studies have focused on interventions aiming to improve social adaptation for people with mental health disorders (58). In line with a growing evidence base on occupational integration for people with mental disorders in general (59), PD interventions with a direct focus on work placement and activity are also in development (60). Such developments underscore occupational activity as a highly relevant outcome when evaluating the course of treatment.

The present study is part of a large, longitudinal, multicenter research project (TREATPD) based on clinical data from the quality register of the Norwegian Network for Personality Disorders (The Network) – a cross-regional collaboration of PD treatment units within specialist mental health services (61, 62). As a whole, TREATPD has the overall aim of providing a broad, longitudinal evaluation of the utility of PD treatment provided within specialist mental health services. It includes longitudinal investigation of clinical outcomes, implementation of specific PD treatments, treatment processes and patient satisfaction, health service use and societal costs, and interactions between the different foci including patterns of concurrent change. In the present study, which is the first TREATPD longitudinal outcome study, a global level of personality functioning is chosen as the main focus, as it constitutes a bridging concept appropriate in a heterogeneous clinical sample. The primary aim is to investigate how personality functioning changes during treatment. In order to supplement the picture of change in personality functioning, the study includes two additional longitudinal outcomes with high clinical and societal relevance: Symptom distress as a frequently included aspect in most studies of treatment outcome, and social/occupational activity as a less studied treatment outcome. Reflecting the likely clinical variation of personality features expanding BPD, a second aim is to specifically investigate differences related to PD conditions frequently presenting in clinical samples.

## Materials and methods

### Design, sample, and treatment setting

The present study is an observational, longitudinal study based on data from the quality register of the Network (61). Data included all patients (*N*_total_ = 1,051) treated at one of the 15 participating Network units in the period 2017–2021. All participating units were situated within outpatient adult specialist mental health and addiction services and specialize on group therapy-based treatments. Their primary target group is people with PDs and personality-related difficulties. Table 1 demonstrates applied treatment approaches. Table 2 demonstrates variation in treatment duration.

**Table 1:** Treatment approaches within the PD services.

	*N* = 1,051	AVPD *N* = 276	BPD *N* = 199	BPD and AvPD *N* = 78	PF NOS *N* = 137	No PD *N* = 314	*N* = 523
Manualized programs	%	%	%	%	%	%	%
MBT	34	28	58	56	29	11	32
DBT	3	3	1	4	0	4	3
SFT	8	6	13	8	5	4	9
MIT	5	10	0	0	0	2	5
Individualized combinations							
Group therapies							
MBT psychoeducation alone	21	23	10	16	24	32	21
Dynamic group	27	31	7	16	34	53	29
Solution-focused	10	12	1	0	12	21	11
Physical training	11	14	3	0	12	21	11
Body awareness	12	14	4	0	7	23	13
Art therapy group	2	14	1	0	15	28	13
Trauma stabilization	1	0	0	0	0	7	1
Social anxiety	0.5	0	0	0	0	2	0.5
Family	5	10	1	0	10	17	6
Other group therapy	11	12	4	0	5	25	12
Combination with individual therapy							
Dynamic	16	13	13	12	17	30	16
CBT	3	5	1	0	2	2	3
Supportive	2	2	0	0	0	2	2
Exposure trauma	0.5	0	0	0	0	2	0.5
Other	6	6	4	0	10	4	6

Table demonstrates the different treatment approaches received in the first 6-month period of treatment for all patients who were referred and completed treatment in the period 2017–2021 (*N* = 1,051), subgroups with different PDs, and the subsample with two assessments or more (*N* = 523). MBT, mentalization-based treatment; DBT, dialectical behavioral therapy; SFT, schema-focused therapy; MIT, metacognitive, interpersonal therapy; CBT, cognitive behavioral therapy.

**Table 2:** Treatment duration and drop-out.

	>36 months %	12–36 months %	6–12 months %	< 6 months %	Drop-out* %	Mean duration (months) (SD)
*N* = 1,051	3	53	28	15	12	15(9)
PD subgroups						
No PD (*N* = 314)	2	41	39	18	9	12 (8)
PD-NOS (*N* = 137)	3	57	28	13	12	15(9)
BPD (*N* = 199)	3	63	26	9	18	17 (9)
AVPD (*N* = 276)	4	59	23	14	10	16 (9)
BPD and AvPD (*N* = 78)	4	71	17	9	17	18 (9)
*N* = 523	6	71	21	2	8	20(9)

Table demonstrates variation in treatment duration for all patients who were referred and completed treatment in the period 2017–2021 (*N* = 1,051), subgroups with different PDs, and the selected study sample for main longitudinal analyses (*N* = 523, bottom row). Differences between PD subgroups were not significant (*p* > 0.05). * Indicates therapist defined drop-out. The rate of treatment dropout was significantly more frequent among patients with BPD (*p* < 0.001).

### Ethics statement

The quality register of the Network includes anonymized clinical data transferred from each treatment unit to the register database. Data collection procedures at each contributing unit are approved by local Data Protection Officers. Security procedures for the quality register are approved by the Data Protection Officer at the responsible research center (Oslo University Hospital). Since data in the quality register are anonymous, formal approvals from the Norwegian State Data Inspectorate and Regional Committee for Medical Research and Ethics are not required. The collection of anonymous, clinical data to the quality register requires informed, written consent.

### Assessment of diagnoses

Participating treatment units applied common routines for diagnostic assessments recommended within the Network. These included semi-structured interviews performed by clinicians at the units, before starting treatment (baseline) – for symptom disorders; the Mini International Neuropsychiatric Interview [MINI (63)], for PDs; the Structured Clinical Interview for section II DSM-5 Personality Disorders [SCID-5-PD (64)]. Clinicians in the Network received training in diagnostic interviews and principles of the LEAD-procedure [Longitudinal, Expert, All-Data (65, 66)]. Diagnostic classification was confirmed by a specialist in psychiatry/clinical psychology at each unit. In the study period, local training courses/workshops focusing on understanding and assessment of PDs, associated comorbidity, use of structured interviews and self-reports were repeatedly conducted in all regions by an experienced psychiatrist and a psychometric expert (first and last authors) at all units in order to ensure clinical competence and calibrate diagnostic evaluation. A total of 29 local workshops were held for clinicians in the period 2017–2019. From 2020, digital seminars were arranged regularly, upon request. In this study we report fulfilled SCID-5-PD diagnoses and the total number of SCID-5-PD criteria (Table 3).

**Table 3:** Demographic and psychosocial status before starting treatment.

	*N* = 1,051		*N* = 523	
	Frequency/scores in clinical range %	Mean (SD) (range)	Frequency/scores in clinical range %	Mean (SD) (range)
Demographics				
Age		30 (9) (17–67)		30 (8) (18–59)
Female	78		78	
Living alone	29		30	
Living with parents	17		15	
Living alone with child	7		7	
Cohabiting/married	33		32	
Years education after mandatory school (age 6–16)		4.2(2)		4.2(3)
Former treatment experience				
Previous treatment in mental health services	82		81	
More than two treatment series	57		58	
First treatment <18 years of age	59		59	
Previous hospital admissions	32		33	
Self-destructive actions				
Self-harming last 6 months	35		35	
Suicide attempt last 6 months	10		12	
Indicators of personality functioning				
Patient-reported personality functioning (LPFS-BF)	80	18(6) (0–36)	86	19(6) (0–36)
Total number of SCID-5-PD criteria		11(6)(0–39)		11(6)(0–39)
No PD	30		27	
PD NOS	15		15	
Specific PD categories				
Schizoid and Schizotypal	1		1	
Paranoid	9		9	
Antisocial	2		1	
Narcissistic and Histrionic	2		2	
Borderline	31		30	
Avoidant	39		41	
Dependent	6		6	
Obsessive compulsive	6		6	
Comorbid symptom disorders and distress				
Patient-reported depressive symptoms (PHQ-9)	90	18(5) (0–27)	93	18(5) (0–27)
Patient-reported anxiety symptoms (GAD-7)	71	13(5) (0–21)	79	13(5) (0–21)
Specific symptom disorders				
Mood disorders	75		79	
Anxiety disorders	47		49	
PTSD	13		10	
Substance use disorder	10		9	
Eating disorder	8		8	
ADHD	7		8	
OCD	4		2	
Somatization disorder	2		3	
Dissociative disorder	0.5		0.3	
Psychosis	0.3		0.3	
Autism	0.2		0.3	
Social and occupational activity				
Patient-reported work/social adaptation (WSAS)	85	24(8) (0–40)	88	24(8) (0–40)
Months in = > 50% work/study last 6 months		2.6 (3.1)		2.7 (3.0)
Months in = > 50% paid work last 6 months		1.0 (2.1)		1.2 (2.3)
Any employment last 6 months (paid work)	23		25	

Table demonstrates demographic, psychosocial and diagnostic status for patients on referral to treatment with separate columns for patients who were referred and completed treatment in the period 2017–2021 (Total sample *N* = 1,051 and subsample with two assessments or more, *N* = 523).

### Battery of evaluation instruments

The battery used in the Network included both patient and clinician reports and was administered at baseline and every 6 months of treatment. Separate items developed for the Network included inquiry (patient self-report) on sociodemographic data (age, gender, family situation, educational level, former treatment experience, self-harming/suicidal incidents). Therapist reports included treatment duration and adherence. The main outcome in this study was: (1) *Personality functioning*: The Levels of Personality Functioning- Brief Form, second version (LPFS-BF 2.0) is a 12-item patient self-report based on the DSM–5 LPFS (Alternative Model of Personality Disorders, Section III) (25) rated on a 0–3 scale, sum-score ranges 0–36. In a German study, population norms (T 50) were at score 15 (67). Correspondingly, in a Danish population study, a total sum-score 0–14 indicated no or minimal dysfunction; >14–18 mild clinical dysfunction; >18 Moderate to severe clinical dysfunction (68). Severe conditions were in this study defined as LPFS-BF > 18 and nonclinical levels as scores <12. Supplementary outcomes included: (2) *Symptom distress*: (a) The Generalized Anxiety Disorder-7 (GAD-7) is a patient self-report of anxiety symptoms with seven items (0–3 scale) (69). Sum scores ≥10 indicate possible anxiety disorder (70). (b) The Patient Health Questionnaire, Depression (PHQ-9) is a patient self-report on depression symptoms (9 items, 0–3 scale), (71, 72). Sum scores ≥10 indicate clinically relevant depressive symptoms (73, 74). In this study severe conditions were defined as GAD-7 > 13 and PHQ-9 values >18, in accordance with mean baseline values. (3) *Social and occupational activity:* (a) The Work and Social Adjustment Scale [WSAS; (75)] is a patient self-report with five items (0–8 scale, 0: no impairment, 8: extreme impairment) reflecting the following social aspects: Ability to work or study, home management, social leisure activities, private leisure activities, and close relationships. Mild-to-no impairment is indicated by sum scores <15, moderate–severe impairment by sum scores 15–30, and extreme impairment by sum scores >30 (76). The WSAS is considered reliable and clinically relevant in a sample comparable to this study sample (77). (b) Separate repeated items developed for the Network included: (i) Patient self-report: The number of months in at least 50% work or study activity last 6 months. (ii) Clinician-rated interview: Enquiry about number of months in paid work last 6 months.

Additional clinician evaluated outcome assessment included a revised version of the Global Assessment of Functioning Scale [GAF (78)], termed the Global Functioning Scale [GFS (79)]. In a study by (80), reliability of the original split-version of GAF was found acceptable (generalizability coefficient: 0.84 for relative decisions, 0.82 for absolute decisions). The GFS gives a single score ranging from 1 to 100, representing symptom severity and social impairment. The lower of the two scores is reported (81). Conventional interpretations of severity are as for the original GAF: Mild (62–71); Moderate (52–61); and Severe (<50). GFS evaluation was performed at baseline and repeated every 6 months of treatment.

### Statistics

All analyses were performed using SPSS Statistics for Windows, Version 27 (82).

#### Longitudinal analyses

Linear mixed models (LMM) based on maximum likelihood statistics were applied (83, 84). Dependent variables based on patient self-report were: (1) *Personality functioning*: LPFS-BF, (2) *Symptom distress*: GAD-7, and PHQ-9, (3) *Social and occupational activity*: WSAS, and number of months in = > 50% work/study last 6 months. Additional longitudinal investigation included the clinical-rated GFS as dependent variable. Time (months from baseline) was modelled as a continuous variable. In accordance with log likelihood estimations, the best-fitted model included linear time, random intercept and slope, and unstructured covariance (critical values for chi-square statistic: *p* < 0.01). Investigation of potential predictors were performed when LMM estimations for the dependent variable indicated significant slope variance (*p* < 0.05). Investigated potential predictors included BPD, AvPD, and total number of SCID-5-PD criteria. Possible variance associated with age and gender was also investigated. PD predictors, age, and gender were investigated first in separate models and then altogether in a final model. We report LMM estimates for change trajectories (intercept and slope), predictor-associated deviation, variance components (intercept and slope), and log likelihood statistics (Akaike’s information Criterion, AIC) (Tables 4, 5). Explained variance is the % reduction of variance estimates from the model without predictor (reference value). In the tables we report exact *p*-values for all values <0.05. Adjustment in accordance with a Bonferroni correction for the four final models would imply statistical inference at the level of *p* < 0.01. Strong inferences are thus indicated by *p* < 0.01 (fixed effects), considered together with % explained variance, and improved model fit (Akaike’s Information Criterion, AIC).

**Table 4:** Longitudinal change and differences according to personality disorder condition.

Model *N* = 466	Predictors	Fixed effects: Estimates for linear trajectories				Variance components		Information criteria
		Intercept estimate (SE)	*p*	Slope estimate (SE)	*p*	Explained intercept variation	Explained slope variation	AIC
Personality functioning								
LPFS-BF		17 (0.2)	<0.001			32**		10,837
LPFS-BF *months		19(0.3)	<0.001	−0.2(0.02)	<0.001	30.8**_(*ref*)_	0.0557**_(*ref*)_	8,009
Separate predictor models	Male gender		ns		ns	1%	1%	8,010
	Age	−0.1(0.03)	0.004		ns	3%	1%	8,002
	BPD	4.5(0.6)	<0.001	−0.11(0.04)	0.009	14%	5%	7,962
	AVPD	1.7(0.6)	0.004		ns	3%	1%	7,996
	PD criteria	0.45(0.04)	<0.001		ns	25%	2%	7,901
Final model	All predictors					29%	7%	7,895
Symptom distress								
GAD-7		12.3(0.1)	<0.001			15**		9,925
GAD-7*months		13.1(0.2)	<0.001	−0.1(0.02)	<0.001	11**	ns	7,420
PHQ-9		16(0.2)	<0.001			21**		10,783
PHQ-9*months		18(0.2)	<0.001	−0.2(0.02)	<0.001	14.0**_(*ref*)_	0.0245**_(*ref*)_	7,921
Separate predictor models	Male gender	−1.9(0.6)	0.001		ns	5%	3%	7,914
	Age		ns	−0.01(0.002)	0.03	1%	4%	7,917
	BPD	1.9(0.5)	<0.001		ns	6%	0%	7,908
	AVPD		ns	0.06(0.04)	ns	0%	5%	7,921
	PD criteria	0.24(0.04)	<0.001		ns	15%	0%	7,869
Final model	All predictors					21%	13%	7,867
Social and occupational activity								
WSAS		21(0.3)	<0.001			44.5**		11,974
WSAS*months		24(0.3)	<0.001	−0.2(0.03)	<0.001	29.9**_(*ref*)_	0.0500*_(*ref*)_	8,873
Separate predictor models	Male gender		ns		ns	1%	11%	8,875
	Age		ns		ns	0%	0%	8,875
	BPD		ns		ns	0%	0%	8,874
	AVPD	2.1(0.7)	0.002	0.10(0.05)	0.06	4%	8%	8,858
	PD criteria	0.3(0.05)	<0.001		ns	13%	0%	8,833
Final model	All predictors					17%	13%	8,828
= > 50% wrk/study		2.6(0.1)	<0.001			5.29**		7,308
wrk/study*months		2.5(0.1)	<0.001	−0.003(0.01)	ns	5.18**_(*ref*)_	0.00500*_(*ref*)_	5,616
Separate predictor models	Male gender		ns		ns	0%	0.2%	5,620
	Age	−0.04 (0.02)	0.02		ns	2%	0%	5,607
	BPD		ns		ns	0%	3%	5,618
	AVPD	−0.82 (0.3)	0.002		ns	4%	4%	5,610
	PD criteria		ns		ns	0%	2%	5,617
Final model	All predictors					5%	0%	5,606

Table demonstrates LMM estimations for patients with at least two assessments, presenting baseline levels (intercept estimate), and monthly change-rate (slope estimate) for 5 dependent variables, separate predictor-analyses for models with significant slope variation, and variance estimates for final models including all predictors together. To enable comparison of models across predictors, the presented estimations are based on a sample including only patients with completed registration of SCID-5-PD (*N* = 466). Non-significant differences are indicated by ns (*p* > 0.05). Significant estimates of variation are indicated by *p < 0.05 and **p < 0.01. Goodness of model fit is indicated by Akaikes Information Criterion (AIC), where smaller is better.

**Table 5:** Longitudinal change and treatment duration.

Model *N* = 1,051	Predictor	Fixed effects: Estimates for linear trajectories				Variance components		Information criteria
		Intercept estimate (SE)	*p*	Slope estimate (SE)	*p*	Explained intercept variation	Explained slope variation	AIC
Personality functioning								
LPFS-BF *months		18(0.2)	<0.001	−0.2(0.02)	<0.001	29.1**_(*ref*)_	0.0552**_(*ref*)_	12,073
Predictor model	Months therapy	0.9 (0.2)	<0.001		ns	2%	0%	12,057
Symptom distress								
PHQ-9*months		18(0.2)	<0.001	−0.2(0.02)	<0.001	14.7**_(*ref*)_	0.0225**_(*ref*)_	11,932
Predictor model	Months therapy	0.6 (0.2)	0.001	0.1(0.03)	<0.001	1%	19%	11,901
Social and work activity								
WSAS*months		23(0.2)	<0.001	−0.2(0.03)	<0.001	30.2**_(*ref*)_	0.0518*_(*ref*)_	13,312
Predictor model	Months therapy	0.6(0.3)	0.004	0.2(0.1)	0.004	1%	6%	13,296
Work/study*months		2.5(0.1)	<0.001		ns	5.54**_(ref)_	0.00645*_(ref)_	8,581
Predictor model	Months therapy		ns		ns	0%	0%	8,584

Table demonstrates LMM estimations (*N* = 1,051) with baseline levels (intercept estimate) and monthly change-rate (slope estimate) for the five models with significant slope variation. Non-significant differences are indicated by ns (*p* > 0.05). Significant estimates of variation are indicated by *p < 0.05 and **p < 0.01. Goodness of model fit is indicated by Akaikes Information Criterion (AIC), where smaller is better.

To further illustrate the magnitude of change, we include calculation of proportions (LMM predicted values) with improvement from start to last phase of treatment, with severe values in the last phase of treatment, with change from clinical to nonclinical score levels, and 0–18 month effect sizes computed according to Cohen’s *d* (Cohen, 1988, small effect size: *d* = 0.2, medium *d* = 0.5, large *d* = 0.8). Eighteen months was an intermediate approximation adjusted to assessment time points (treatment duration ≤ 18 months: 67%).

#### Number of repeated measurements

Mean number of repeated assessments in the total sample (*N* = 1,051) was 1.9 (SD 1.1, min 1 and max 8). Among these, a total of 523 individuals had two assessments or more (mean 2.8, SD 1.0, at least three assessments: 47%). Baseline status in the total sample (*N* = 1,051) and the subsample of 523 individuals with two or more assessments was similar (Table 2). Mean treatment duration was longer for the subsample (*N* = 523, Table 3). Longitudinal analyses included subsample models (*N* = 523) and replication in total sample models (*N* = 1,051).

#### Missing data and unbalanced data

Missing data for the quality register were due to different locally occurring, administrative failures of delivery or registration. The data-collection for the quality register was limited to the treatment period. The data were therefore unbalanced as shorter treatment duration naturally caused fewer assessments. To investigate possible systematic bias of missing data, a variable counting the number of assessment points was investigated as a longitudinal predictor in separate models for all dependent variables (85). This missing data analysis is presented in the results section. As treatment duration varied within the sample its associations to outcomes was also investigated as a separate predictor in LMMs based on the total sample (*N* = 1,051, Table 5).

#### The COVID-19 pandemic

The majority of patients were enrolled before the Covid-19 pandemic (from March 2020). Delivery of self-reports from patients did not change during the pandemic period after March 2020 (*p* > 0.05). Due to conceivable inflation of symptom distress and functional impairment in this period (86), and the change in treatment situation during the first pandemic wave (March–June 2020) (87) we include investigation of possible effects on the longitudinal course of PHQ-9 and WSAS. A dichotomous variable distinguishing subsamples with pre and post pandemic treatment completion was investigated as a predictor in WSAS and PHQ-9 models (85). A time-variable with two time points (treatment start and last phase) was used as treatment duration was a notable difference between the two subsamples. Results are presented in the results section.

#### Other descriptives

These included chi-square tests, Mc Nemar-Bowker Test, independent samples *T*-test, and paired samples *T*-test.

## Results

### Status on treatment referral

Table 3 presents baseline descriptive data on demographic status, prior treatment experience, self-harming behaviors, diagnoses, work/study activity together with scores of LPFS-BF, PHQ-9, GAD-7, and WSAS. The majority were females in a young adult age group (58% < 30 years), 70% qualified for at least one PD, and a notable proportion had more than one PD (24%). Mean number of SCID-PD-5 criteria for patients with PD was 12.9 (SD 5.7) and mean number of criteria for patients below PD threshold, 3.5 (SD 2.4). The most common PDs were AvPD and BPD.

### Longitudinal change during treatment

**Figure 1:**
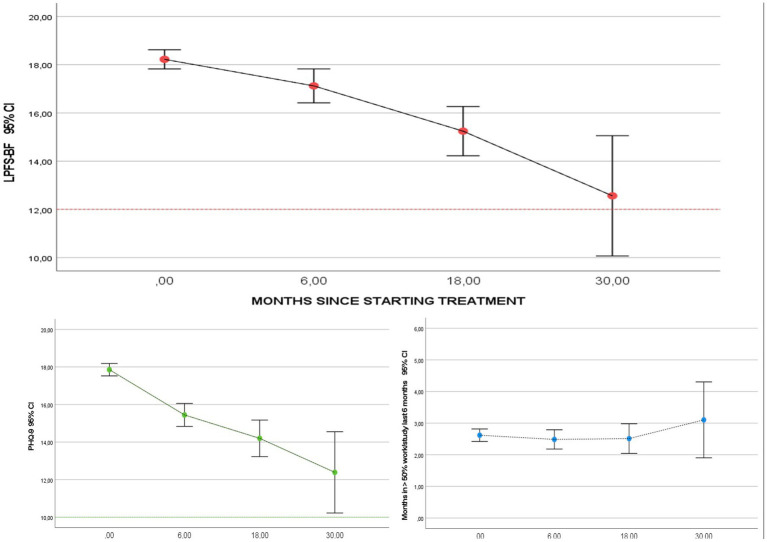
Longitudinal change during treatment. Figure demonstrates personality functioning (LPFS-BF), symptom distress (PHQ-9), and occupational activity (= > 50% work/study) assessed at different time-points during treatment (*N* = 1,051). The *Y*-axes indicate scores of the LPFS-BF, PHQ-9 and number of months in occupational activity. The *X*-axes indicate the time-points of assessment during treatment. Within subject variation is demonstrated by calculated confidence intervals (CI).

**Figure 2:**
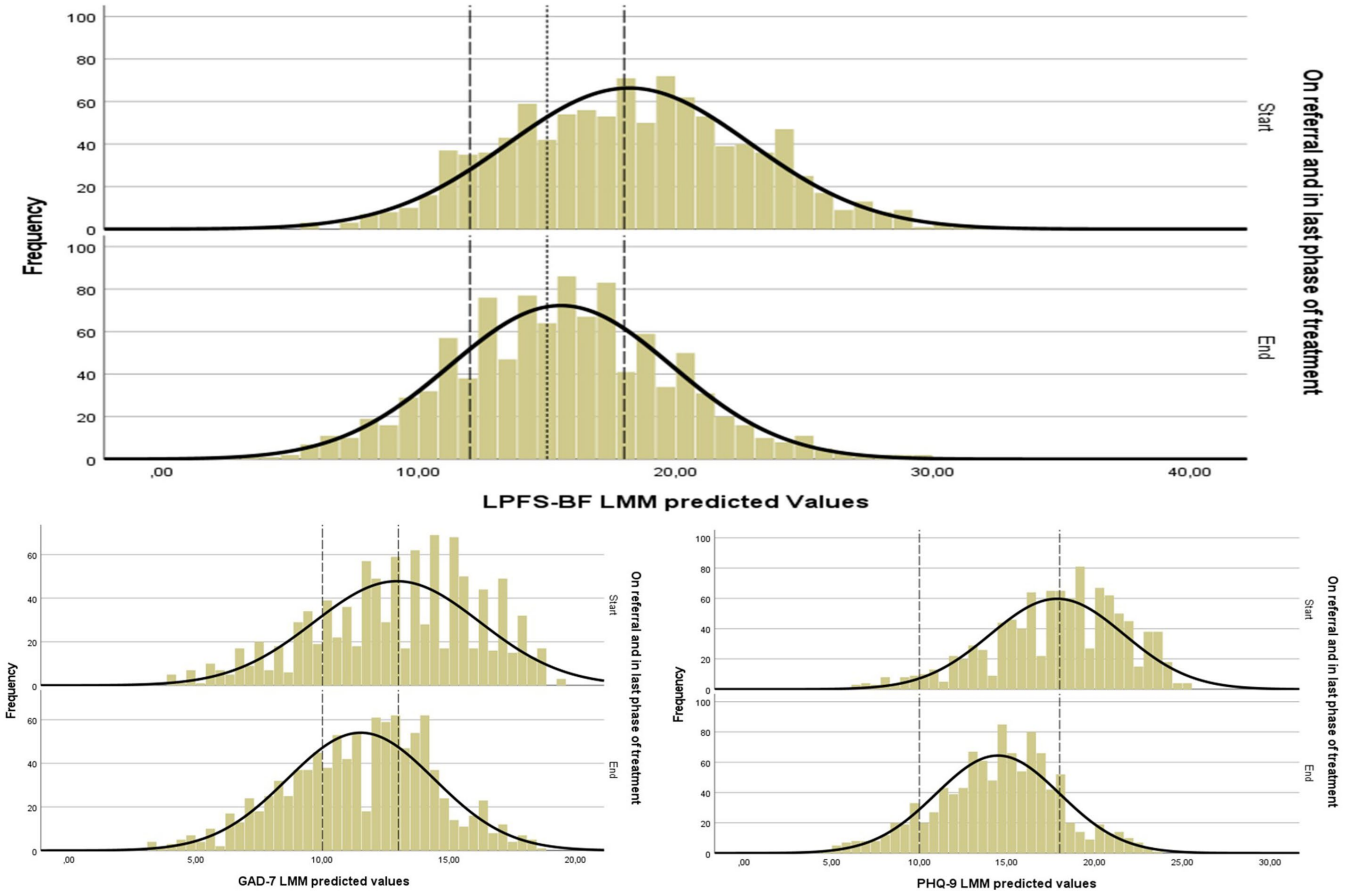
Distribution of scores of LPFS-BF, PHQ-9, and GAF Figure demonstrates the distribution of scores of personality functioning (LPFS-BF), and symptom distress (PHQ-9, GAD7) assessed on referral and in the last phase of treatment. The figure presents LMM predicted values based on the total sample (*N* = 1,051). Dashed lines to the left indicate clinical-nonclinical levels and to the right scores of considerable severity. Dotted line indicates LPFS-BF norm in a general population study (67).

LMM analyses (samples *N* = 523 and *N* = 1,051) rendered significant trajectories of longitudinal improvement for LPFS-BF, as well as PHQ-9, and GAD-7 (Table 4; Figure 1) with estimated 0–18-month effect sizes in the moderate to high range (LPFS-BF: ES 0.7, PHQ-9: ES 1.4, GAD-7: ES 0.6, mean ES: 0.9). Figure 2 gives the distribution of scores at referral and last phase of treatment. Among patients with scores in a clinical range at the start of treatment, large proportions had severe level scores (LPFS-BF > 18: 57%, PHQ-9 > 18: 53%, GAD7 > 13: 63%, total mean 58%). From start to last phase of treatment the majority indicated a score reduction (LPFS-BF: 64%, PHQ-9: 66%, GAD7: 54%, total mean 61%). The mean proportion with severe values in the last phase of treatment (indicative of poor response) was 24% (LPFS-BF > 18: 26%, PHQ-9 > 18: 13%, GAD-7 > 13: 32%). The mean proportion which moved from clinical to nonclinical values (indicative of recovery) was 12% (LPFS-BF < 12: 9–22%, PHQ-9 < 10: 4–12%, GAD-7 < 10:19–31%, overall mean 10–22%). Analyses of social and occupational activity rendered a mixed picture with overall significant improvement of WSAS, but not significant linear change of work/study activity (Table 4; Figure 1). Results were replicated in the total sample (*N* = 1,051, WSAS 0–18 months ES: 1.0, months = > 50% work/study ES: 0). In the last phase of treatment, mean number of months in = > 50% work/study last 6 months was 2.7 (SD 2.9, range 0–12), and among patients with no paid work last 6 months before referral (*N* = 784), 86% still lacked regular employment (change from start to end of therapy: *p* = 0.11).

In additional investigation of clinician-rated change, LMM analyses rendered trends of severe initial levels (GFS < 60), and significant improvement of GFS over time (LMM estimates: mean intercept GFS 52(SE 2), mean slope 0.2 (SE 0.02), *p*_slope_ < 0.001).

### Analyses of predictors

Slope variation was significant in models with LPFS-BF, PHQ-9, WSAS and work/study activity. Thus, for these variables, patients’ clinical change trajectories were highly diversive. Slope variation was not significant in GAD-7 models (Table 4).

#### Personality functioning (LPFS-BF models)

**Figure 3:**
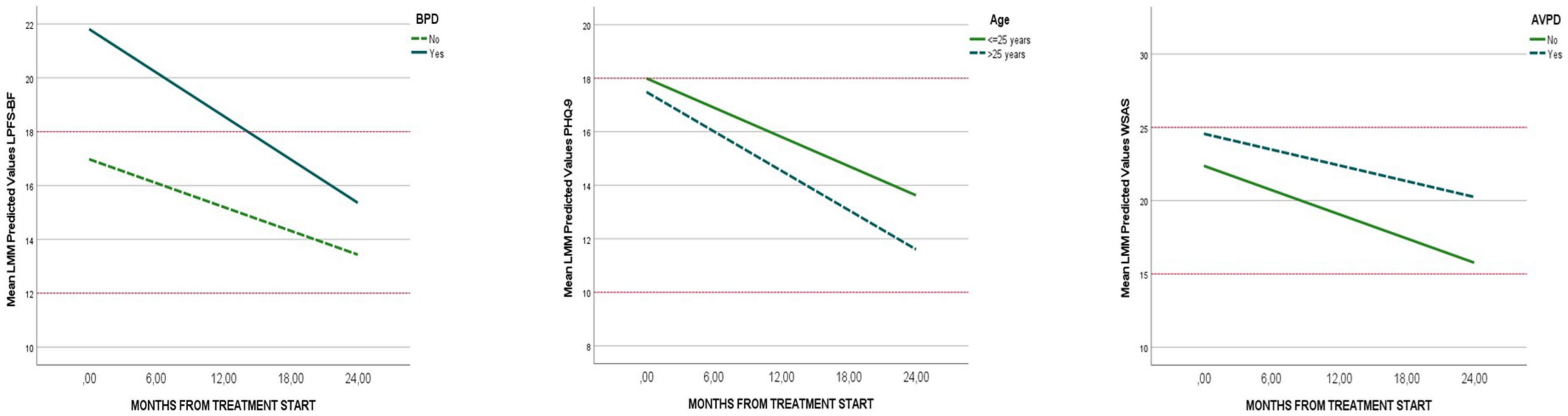
Demonstrates longitudinal trajectories (LMM predicted values, *N* = 1,051) for significant predictors of personality functioning (LPFS-BF), symptom distress (PHQ-9), and work and social activity (WSAS). The *Y*-axes indicate scores of the LPFS-BF, PHQ-9 and WSAS. The *X*-axes indicate the time-points of assessment during treatment.

All PD predictors (BPD, AvPD and higher number of total PD criteria) were significantly associated with more impaired baseline LPFS-BF (Table 4). Over time, only BPD was a significant longitudinal predictor, associated with more rapid LPFS-BF improvement compared to patients without BPD; a reduction of 0.31 LPFS-BF points per month was estimated for BPD versus a reduction of 0.2 LPFS-BF points for the rest of the sample (Table 4; Figure 3). Results were evident in separate predictor models and final models with all potential PD predictors, age and gender included (explained intercept variation: 29%, explained slope variation: 7%). In total sample models (*N* = 1,051), intercept and slope results were replicated for BPD, and intercept results were significantly replicated for total number of PD criteria and age, but not AvPD (*p* > 0.05).

#### Symptom distress (PHQ-9 models)

Among predictors, higher number of total PD criteria and female gender were associated with more severe initial levels of PHQ-9. Over time, no PD predictors were significant. Higher age was moderately associated with more rapid PHQ-9 improvement (Table 4; Figure 3). These results were evident in separate predictor models, in final models including all potential PD predictors, age and gender included (explained intercept variation: 21%, explained slope variation: 13%), and in total sample models. Table 4 also demonstrates separate intercept effects of BPD. This trend was neither maintained in the final model nor replicated in the larger total sample (*p* > 0.05).

#### Social and occupational activity

##### Work and social adjustment scale models

AvPD and total number of PD criteria were associated with poorer initial levels of WSAS (Table 4, separate predictor models, models with all PD predictors, age and gender, and total sample models). Over time, AvPD was in separate models modestly (*p* = 0.06, explained slope variation 5%) associated with less WSAS improvement compared to patients without AvPD, i.e., a reduction of 0.1 WSAS points per month was estimated for AvPD versus a reduction of 0.2 WSAS points for the rest of the sample (Table 4; Figure 3). In the final model including all potential PD predictors, gender and age, the longitudinal effects of AvPD were stronger (*p* = 0.03, explained intercept variation: 17%, explained slope variation: 13%). Results were replicated in total sample models.

##### Models with work/study activity

AvPD and younger age were associated with fewer months in work/study activity before starting treatment (Table 4). None of the investigated potential predictor variables were associated with deviating change (Table 4). Results were evident in separate predictor models, in final models including all potential PD predictors, age and gender (explained intercept variation: 5%, no further explained slope variation), and in total sample models.

#### Treatment duration

**Figure 4:**
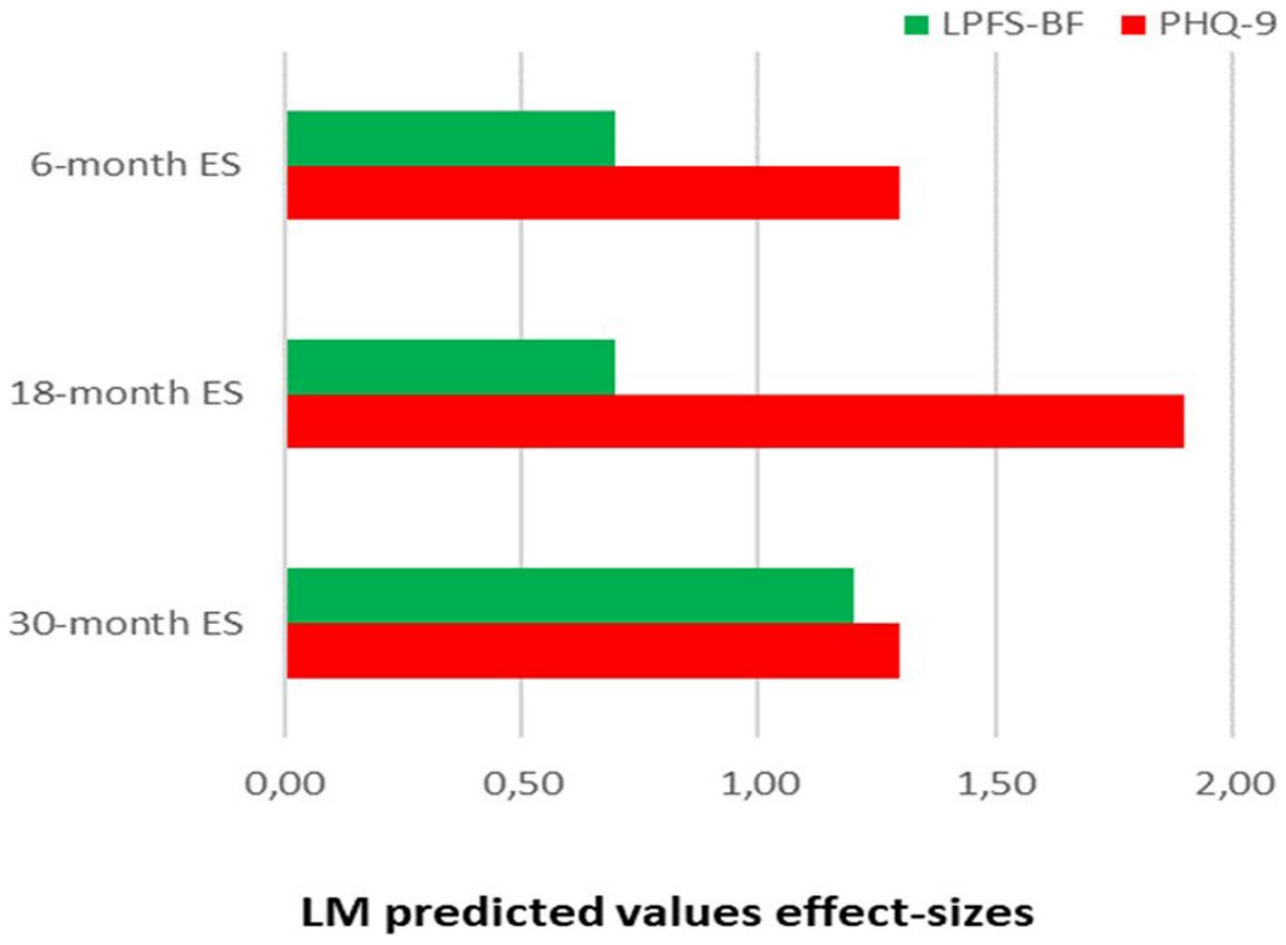
Demonstrates LMM 6 month effect sizes (ES) for patients with 0–9 months duration (30%), 18 month effect sizes for patients with 9, 1–18 months duration (37%), and 30 month ES for patients with >18 months duration (33%) 0.

Mean treatment duration in the total sample (*N* = 1,051) was 15 months (SD 9, Table 2). Longer treatment duration was significantly associated with having more severe conditions on referral (LPFS-BF, PHQ-9, WSAS, Table 5). Largest proportion intercept variation was explained by LPFS-BF (2%). Rates of LPFS-BF improvement did not differ by treatment duration (Table 5). Longer treatment duration was associated with significantly slower rates of PHQ-9 and WSAS improvement (Table 5). Explained variation was largest for PHQ-9 (19%). Differences in treatment duration were not associated with deviating initials levels and improvement of work/study activity. Figure 4 illustrates differences by estimated effects sizes (ES) based on linear change for different treatment durations. In the briefest treatments (< 6 months), the LPFS-BF and WSAS ES was <1, whereas the PHQ-9 ES was >1. In treatments with long duration the LPFS-BF and WSAS ES was >1.

#### Missing data analyses

The number of assessment points were investigated as a separate longitudinal predictor in the total sample (*N* = 1,051). Higher number of assessments was not associated with a deviating change pattern for any of the dependent variables (*p* > 0.1).

#### COVID-19 pandemic

In analyses differentiating pre-and post-pandemic subsamples, LMM longitudinal trends were comparable, and no significant differences in end points were found (*p* = 0.89).

## Discussion

This study uniquely describes the longitudinal course of personality functioning along with essential aspects of symptom distress and social activity in a large sample of patients treated within regular specialized PD mental health services. As could be expected, on referral, the present sample is characterized by considerable psychosocial burden, it is dominated by patients with PD, but heterogeneous with respect to PD type and severity. As described in Table 1, the treatments applied in the Network units included both long-term psychodynamic group therapies, a variety of shorter-term treatments, and evidence-based BPD treatment programs, among which components of mentalization-based treatment (MBT) were the most frequent. In line with other research, initial severity of all the investigated aspects (personality functioning, symptom distress, and social and occupational activity) was strongly related to PD status, both PD presence and severity as measured by the number of fulfilled PD criteria across categories (10, 24, 88). The main longitudinal trends during treatment are summarized in the following: (1) Significant improvement of personality functioning and symptoms with differences associated with PD status. (2) Contrastingly, poor improvement of occupational activity. (3) The study reveals noteworthy age-related differences in initial status and change over time.

### Improvement of personality functioning

The potential for improvement of personality functioning during treatment is a primary research focus in this study, as it is in treatment for people with PD. Early studies of comparable heterogenic PD samples receiving psychodynamic group-based treatments naturally, lack reports on change of personality functioning domains (10, 88, 89). In both clinical trials and naturalistic studies of BPD treatments, main outcomes often include high-risk actions such as self-harming and suicide attempts, the need for hospital admissions, and improvement of specific BPD traits (6, 38, 90, 91). More recent studies of BPD treatment report moderate to large effect sizes for different personality functioning aspects (92–95). In our study, including a large sample, a broader range of PDs, and heterogeneity of treatment intervention, we also demonstrate overall trends of improvement with a moderate to large effect size for personality functioning together with declining levels of symptom distress. In line with the large effect sizes reported in other referred BPD studies, we found BPD associated with particularly high improvement rates, starting from the most severe levels.

Personality functioning has been advanced as an indicator for clinical decision making (28, 96, 97). Our data do indeed suggest a systematic selection of patients with poorer personality functioning to treatment with longer duration. Moreover, results also indicated greater improvement of personality functioning for patients with longer time spent in treatment. It is possible that further investigation of other treatment factors could have explained more outcome variation in the sample. The brief overview of the applied treatment approaches within the Network demonstrates that evidence-based, long-term BPD treatments were quite frequently applied across PDs, in particular for patients with BPD and AvPD-BPD. Such treatments keep an explicit psychotherapeutic and didactical focus on core aspects of personality functioning (98). Studies of mentalization-based treatment (MBT) have suggested treatment potentials for people with severe conditions expanding the single diagnoses of BPD (99–101). Within the Network, group psychotherapy is a central aspect in all the PD treatments provided, but composition of programs, theoretical approach, implementation of manualized PD treatments and treatment duration varies according to patients and local resources. More detailed investigations and conclusions concerning treatment factors, such as style of intervention, format or intensity of treatment, treatment processes and association to outcomes are outside the scope of the present study. Further research specifically focusing on the utility and application of specialized, manualized PD treatments in heterogenic clinical samples is scheduled within the TREATPD project.

### Drop-out rates were low

Rates of therapist defined drop-out from treatment were generally low in our study although somewhat higher for patients with BPD and comorbid PDs. For comparison, other reviews report considerably higher mean drop-out rates (27%) for patients with BPD (102, 103).

Achieving best possible treatment adherence is essential in psychotherapy for patients with PD. Treatments designed for PD often focus on key features of personality functioning [Laurenssen et al., 2014; (25), such as emotional dysregulation (104, 105), awareness of affects (106), mentalizing and interpersonal functioning (107), and in PD therapies, systematic co-creation of individual case formulations addressing such problems is recommended (108)]. Use of pedagogical interventions and psychoeducation may also strengthen commitment and motivation in treatment for PD (109–111). In our sample, approximately 50% received psychoeducation as part of a manualized program, and additionally 21% attended a standalone MBT psychoeducation.

Alliance may be reinforced by concomitant feedback-systems monitoring the process (112). Seeking to support explicit, individually tailored, personality focus and alliance during treatment, a feed-back system with graphical profiles reflecting patients’ half-yearly self-reports on progress, is available for all therapists in the Network, and is recommended as supplementary information for clinical reviews and patient-therapist dialogues (61). In the current sample, such systems may have facilitated better understanding of the individual patient and the ongoing process, and thus also contributed to treatment adherence. In a study of alliance in MBT, therapies with good outcomes were characterized by particularly positive development of the mutual understanding of tasks in therapy (113). Further research specifically investigating alliance in treatment of PD is warranted.

### Improvement, poor response, and recovery

Although the study demonstrates a main trend of improvement across outcome measures, variation is to be expected. The majority reported improvement, also patients with initially severe conditions. However, few reached non-clinical score levels of personality functioning and symptoms. Complete recovery to strictly defined, non-clinical score levels in the last phase of treatment, was only indicated for 12%. The opposite, a clinically severe condition at the end of treatment, was indicated for 24%. For comparison, a former five-year follow-up study of a corresponding clinical mixed PD sample treated in the period 1992–1998, reported 36% with persisting symptom distress after group-based treatment (10). Moreover, a recent review of 10 BPD psychotherapy studies, indicated 49% non-response rates (103). However, the authors comment considerable heterogeneity of definitions of non-response, and generally a lack of studies reporting and nuancing poor response to treatment interventions. In the present study, our results indicate an overall majority with improvement, but nonetheless, a main trend with persistence of some problems on levels of clinical relevance and 24% at the extreme end. For future research, poor responders are important to identify. With health services generally tending toward increasing specialization, patients with complex conditions and comorbid disorders are at risk of falling between health services (114).

### Poor occupational activity

As one of few treatment studies, we have included specification of occupational activity. At the start of treatment 77% had not been in paid work the last 6 months. We regret to conclude that work/study activity was generally and persistently poor across PDs despite treatment, and it is noteworthy that different PD conditions explained minimal longitudinal variation. Nuances in the development of work/study activity may have been lost due to the limited detail of this variable. However, trends were also confirmed by the interview-based variable counting months in paid work which included any part time proportion.

An imbalance between improvement of PD features and occupational functioning has been described in former psychotherapy studies and large longitudinal studies of PD populations (115–118). The cited US based longitudinal studies have described that clinical PD samples tend to improve a lot, but more so in terms of symptoms and PD traits than in occupational functioning. In our study of a large Norwegian sample, despite differences concerning both work and social welfare systems, we nonetheless, and correspondingly, found that clinical improvements lacked reflection in occupational activity. The findings were evident across PD conditions. Compared to a Norwegian MBT study recruiting only BPD patients from an urban area (119) the present cross regional sample recruited patients from both urban and rural areas and reported persistently poorer work status from the start. For patients in intensive psychotherapy programs, such results may to some extent be related to possible difficulties combining work and therapy. Follow-up investigations could have provided better reflection of the patients’ capacity and work possibilities after ended treatment. Other follow-up studies of BPD patients have suggested potentials for moderate long-term occupational improvement – 39% with remitted occupational impairment after 20 years (5), and in an 8 year follow-up after MBT, a 53% likelihood of employment (55).

Finding employment and staying employed are known to be difficult for people with emotional and relational problems, both PDs and subthreshold PD traits (5, 120), and relevant issues include diverse expectations to the work situation or capacities of the work place as well as the person’s management of own personality problems (4, 51, 121). Interdisciplinary cooperation between mental health and public welfare services can be essential when engaging patients in the workforce. Recent studies advocate employment supporting interventions integrated with treatment for people with PDs (122). The present results point to a need for more focused developments and research.

### Restricted social engagement among patients with avoidant personality disorder

The course of social activity varied within the sample and compared to patients without AvPD, patients with AvPD were associated with slower change in the overall measure of work and social adjustment. WSAS items include not only work functioning, but also engagement in social activities and closer relationships – obviously picking up core challenges for people with AvPD. Although LPFS-BF also includes interpersonal items, AvPD was not a correspondingly, significant predictor of the longitudinal course of personality functioning. However, the analyses revealed a small proportion explained slope variation suggestive of some impact. The possible discrepancies between WSAS and LPFS-BF may be due to psychometric qualities of the instrument. A recent study examining the construct validity of the self-report LPFS-BF in a Norwegian sample, described low sensitivity for AvPD (123).

AvPD was the most frequent PD in our clinical sample and the trend of moderate WSAS improvement is in line with former comparable studies from earlier time periods (12, 88). The deviating trends associated with AvPD in our study were mainly evident for social activity aspects and not significant for other outcomes. Improvement of symptom distress in the present study seems better than demonstrated for patients with AvPD in a former study of traditional group-based psychodynamic treatments (10, 124). Although research and treatment developments targeting this patient group are in progress (125–127), studies on AvPD features are still underrepresented (128). Our study specifically points to the pattern of prevailing restricted social engagement which may require more tailored intervention.

### Age-related poor personality functioning

In line with other studies (129, 130), we found younger age associated with more impaired personality functioning. We also found moderate indications of poorer work/study activity and more persisting depressive symptoms. Other PD studies have comparatively, associated younger age with more complex symptoms, comorbid PD conditions, more dysfunctional coping strategies, and poorer treatment outcomes (129, 130). Taken together, these findings may imply that younger patients need an adjusted treatment approach. Specialized treatments for adolescents with BPD are established (131) and also in development for young people with AvPD (126).

### Concurrent change

*C*omparing improvement in personality functioning and the supplementary outcome measures, our study suggests different patterns of change. In our study results suggest that depressive symptoms may generally remit more readily (large effect sizes evident also in treatments of short duration), whereas personality functioning and social activity (WSAS), improved at slower rates, reaching notable effect sizes in long-term treatments. Further analyses of concurrent change was not included in this study. However, such analyses can provide clinically relevant information on complex mechanisms of change in treatment for PD. In a study of BPD patients (N = 50), their expression of global distress in the first phase of therapy, predicted positive interpersonal outcomes later in therapy, given a responsive therapeutic interaction (132). Moreover, a former study based on a similar, mixed PD sample (*N* = 113) indicated interrelated change of personality and psychosocial functioning (133). In this study improvement of relational aspects of personality functioning was found to precede global psychosocial improvement.

### Strengths and limitations

The present study is based on a data collection enabled by the collaborative quality and research network advancing common procedures for PD assessment and running treatment evaluation at PD treatment units across health regions. A major strength of the current study is its reflection of regular clinical practice as implemented and conducted within specialist mental health services. The sample is uniquely large and research data include detailed information relevant for the study of PD.

As regular clinical practice, diagnostic procedures in Network units generally hold a high standard as they are based on structured interviews and conducted by qualified health professionals. It is nonetheless a limitation that diagnostic reliability was not investigated.

It is a possible limitation that clinical outcome measures were based on patient-self-report. We therefore included additional analyses of clinician-rated global functioning (GFS) which indicated corresponding longitudinal trends as found in the patient self-report. The contrasting results of persisting work impairment despite clinical improvement may indicate a discrepancy between measures and real-world functioning (134). However, the included measures of activity did include both clinician-performed interviews and patient self-reports. Trends for work activity reported by self-report versus therapist interview did differ.

Incomplete data sets represent a limitation to conclusions. Missing data are to be anticipated in naturalistic treatment settings with repeated assessments over long time periods, and paper-based administration of questionnaires and registration of data is vulnerable. The chosen statistical method is appropriate for samples with unbalanced data and long study periods and enables utilization of available longitudinal data (84). In order not to overestimate trends, we report LMM analyses of the subsample with more frequent measurement occasions (*N* = 523) as well as analyses of the total sample (*N* = 1,051) and include consideration of possible missing data patterns (85).

Being based on available data from a quality registry, our study lacks post treatment evaluations. As a possible consequence, longitudinal trends may have been underestimated. Several studies of group-based treatments demonstrate significant developments after ended psychotherapy (135, 136).

## Conclusion

This study investigates a clinically representative sample of patients with different PD conditions enrolled in PD treatment of varying approach and format within specialist mental health services. Its primary aim was to investigate how personality functioning changes during treatment. The study demonstrates a main trend of improvement across PD conditions. However, in sharp contrast to the general improvement of personality functioning and symptom distress, the study demonstrates that occupational activity was persistently poor. By investigating differences associated with PD conditions, the study in particular highlights improvement potentials for BPD patients with severely impaired personality functioning. The study points to age-related differences in personality functioning and indicates a need for further developments and research addressing social disengagement among patients with AvPD.

## Data availability statement

The datasets presented in this article are not readily available because the data used in this study are based on a quality register of the Norwegian Network for Personality Disorders. Due to restrictions regarding patient confidentiality, data are only available on specific request. Requests may be sent to the Privacy and Data protection Officer at Oslo University Hospital; personvern@ous-hf.no. Requests to access the datasets should be directed to personvern@ous-hf.no or corresponding author, EK e.h.kvarstein@medisin.uio.no.

## Ethics statement

Ethical approval was not provided for this study on human participants because the quality register of the Network includes anonymized clinical data transferred from each treatment unit to the register database. Data collection procedures at each contributing unit are approved by local Data Protection Officers. Security procedures for the quality register are approved by the Data Protection Officer at the responsible research centre (Oslo University Hospital). Since data in the quality register are anonymous, formal approvals from the Norwegian State Data Inspectorate and Regional Committee for Medical Research and Ethics are not required. The patients/participants provided their written informed consent to participate in this study.

## Author contributions

EHK, the first and corresponding author, was responsible for data analyses and manuscript development together with MF. BH, TW, IU-ME, KTEM, DAU and GP have contributed in discussion of analyses and presentation. GP is the last author, responsible for the Network collaboration, current data collection, and data preparation. All authors have contributed to design and/or data collection and manuscript revisions.

## Funding

Oslo University Hospital, University of Oslo, and Akershus University Hospital have supported the study.

## Conflict of interest

The authors declare that the research was conducted in the absence of any commercial or financial relationships that could be construed as a potential conflict of interest.

## Publisher’s note

All claims expressed in this article are solely those of the authors and do not necessarily represent those of their affiliated organizations, or those of the publisher, the editors and the reviewers. Any product that may be evaluated in this article, or claim that may be made by its manufacturer, is not guaranteed or endorsed by the publisher.
